# Public understanding of preprints: How audiences make sense of unreviewed research in the news

**DOI:** 10.1177/09636625241268881

**Published:** 2024-10-11

**Authors:** Alice Fleerackers, Chelsea L. Ratcliff, Rebekah Wicke, Andy J. King, Jakob D. Jensen

**Affiliations:** The University of British Columbia, Canada; Simon Fraser University, Canada; University of Georgia, USA; Cornell University, USA; The University of Utah, USA; Huntsman Cancer Institute, USA

**Keywords:** COVID-19, journalism, preprints, public understanding of science, scientific uncertainty

## Abstract

News reporting of preprints became commonplace during the COVID-19 pandemic, yet the extent to which the public understands what preprints are is unclear. We sought to fill this gap by conducting a content analysis of 1702 definitions of the term “preprint” that were generated by the US general population and college students. We found that only about one in five people were able to define preprints in ways that align with scholarly conceptualizations of the term, although participants provided a wide array of “other” definitions of preprints that suggest at least a partial understanding of the term. Providing participants with a definition of preprints in a news article helped improve preprint understanding for the student sample, but not for the general population. Our findings shed light on misperceptions that the public has about preprints, underscoring the importance of better education about the nature of preprint research.

Preprints offer a way for researchers to share new discoveries with other researchers more quickly than through traditional peer review, making them a valuable communication tool—especially during rapidly evolving situations or crises (Johansson et al., 2018). Yet, journalists have historically been discouraged from reporting on preprints due, in part, to fears that such coverage could spread uncertainty or misinformation (Froke et al., 2020; Sheldon, 2018a). As research papers made publicly available before journal peer review (Berg et al., 2016), preprint conclusions can sometimes change between initial posting on a preprint server and later publication in a journal. While most preprints closely resemble their published versions, a proportion change in important ways (e.g. reversal of conclusions or introduction of new findings) (Brierley et al., 2022; Nicholson et al., 2022) or are never published at all (Drzymalla et al., 2022). However, during the early months of the COVID-19 pandemic, preprint-based journalism surged (Fleerackers et al., 2024b; Fraser et al., 2021), with preprints receiving media coverage in countries such as Canada, the United States, South Africa, Brazil, Germany, and the United Kingdom (Fleerackers et al., 2022b; Massarani and Neves, 2022; Oliveira et al., 2021; Simons and Schniedermann, 2023; Van Schalkwyk and Dudek, 2022).

Although preprint-based journalism may become a “new normal” (Fleerackers et al., 2022a), journalists still lack evidence-based best practices for communicating the unreviewed nature of these studies in ways that audiences can understand (Ginsparg, 2021). In addition, it remains unclear how audiences conceptualize preprints and what factors might help shape these conceptualizations (Fleerackers et al., 2024a). This study addresses this gap through a content analysis of 1702 definitions of the term *preprint* generated by samples of the US general population and students. The study examines the degree to which these definitions of preprints align with those of scholars (RQ1a) and describes alternative conceptualizations that are common among public audiences (RQ1b). We also examine factors that may contribute to greater public understanding of preprints, including exposure to preprint definitions in the news (RQ2a) and participant characteristics (e.g. age, education, factual scientific literacy) (RQ2b).

## 1. Literature review

Preprints have been a small but growing part of scholarly communication for more than 30 years (Puebla et al., 2022), with the number of preprints doubling approximately every decade (Xie et al., 2021). Yet, before COVID-19, little was known about how and why journalists or their audiences engage with these unreviewed studies (Fleerackers et al., 2024a). The scant scholarly literature from before the pandemic comprised mainly opinion pieces (e.g. Fraser and Polka, 2018; Sarabipour, 2018) and interview-based studies examining the scholarly community’s perceptions of public communication of preprints (e.g. Chiarelli et al., 2019), rather than empirical studies investigating how and why journalists cover these unreviewed studies or how their audiences perceive them. Collectively, these contributions highlighted growing concerns among scholars, journalists, and science communicators about the potential for preprint research—if covered widely by the media—to “undermine measured and accurate communication of science” and have “possible unforeseen effects on the public’s understanding of science” (Sheldon, 2018b: 1194).

Despite these concerns, research into preprint-based journalism did not emerge until after the onset of the pandemic, when widespread coverage of these unreviewed studies revitalized concerns about their potential to spread inaccurate information (Majumder and Mandl, 2020; Van Schalkwyk et al., 2020). These concerns prompted calls for more research examining the public communication of preprint research (Caulfield et al., 2021), as well as for the development of “protocols” and “guidelines” for communicating preprints’ unreviewed nature and any associated uncertainties in ways that public audiences can understand (Brierley, 2021; Kardos et al., 2023). To some extent, these calls were heeded by professional journalism and scholarly communication organizations, many of which published blog posts and tip sheets recommending that journalists communicate the preprint nature of the research and stress that it has not yet been peer reviewed (ASAPbio, n.d.; Van Schalkwyk and Dudek, 2022). Perhaps as a result, communicating a study’s preprint status became an accepted aspect of accurate and ethical science communication both among scholars (Kardos et al., 2023; Wingen et al., 2022) and professional journalists (Fleerackers et al., 2022a; Schultz, 2023). Indeed, much of the early media coverage of COVID-19 preprints included statements emphasizing that the research in question had not yet been peer reviewed or had been posted as a preprint (Fleerackers et al., 2022b; Massarani and Neves, 2022; Oliveira et al., 2021; Van Schalkwyk and Dudek, 2022).

Yet, even as journalists, media organizations, and scholars urged communicators to acknowledge preprints’ unreviewed status, others have voiced concerns about the effectiveness of this approach (Cataldo et al., 2023; Fleerackers et al., 2022a). For example, journalists have questioned whether disclosures of a study’s preprint status are understood by audiences, even though journalists believe disclosures are important to include (Fleerackers et al., 2022a). Researchers have similarly argued that non-expert audiences likely do not understand what preprints are (Kardos et al., 2023; Simons and Schniedermann, 2023), echoing a long-standing belief that the concept of peer review is not well understood by those outside the academic community (Sense About Science, 2009). Others remained undecided, urging researchers to “consider what ‘peer review’ has meant for decades to the nonscientist taxpaying public” (Berenbaum, 2023: 2) and calling for greater efforts “to gauge how well the research community and the public understand the limitations of preprints” (Gopalakrishna, 2021: para. 7).

The conflicting perspectives on public understanding of preprints is unsurprising given limited empirical research in this area. Preliminary insights can be gleaned from Song et al. (2022), who found that public audiences could identify research conducted using open science practices when these practices were made clear in a format similar to a news story. Audience members who were able to identify the open science practices also perceived the research to be more trustworthy and of higher quality than research that had not been described as using such practices. However, the study did not examine the use of preprints specifically, which may be perceived or understood differently than other forms of open science (e.g. preregistration, open data, open access journal publishing). More specific to preprints, a series of studies with high school, community college, undergraduate, and graduate students found that very few students (of any education level) successfully differentiated preprint research from peer-reviewed articles, with many noting that they did not understand the term *preprint* or its connection to peer review (Cataldo et al., 2023; Cyr et al., 2021).

Similarly, across five different samples, Wingen et al. (2022) found that publics did not perceive a difference in the credibility of preprints and peer-reviewed papers—results that held true even when the research was accompanied by a brief statement that preprints are studies that have not been peer reviewed. Only when that brief statement was accompanied by a lengthier description of the peer review process did participants perceive the research to be less credible—suggesting that the terms *preprint* and *peer review* have little meaning to public audiences as standalone terms. Finally, a recent study by our team found that only about 10% of a US audience was able to define the term *preprint* in ways consistent with scholars’ understanding of the term, with an additional 15% understanding it as indicating preliminary evidence (Ratcliff et al., 2024). However, the other three quarters of that sample either did not know or provided an alternative definition of the term that we classified as “other” and did not analyze in detail. Moreover, seeing a definition of the term *preprint* in a news story did not influence the accuracy of audience members’ definitions (Ratcliff et al., 2024).

Other than Ratcliff et al. (2024), no existing studies have directly examined how publics understand preprints, as well as what factors, if any, may help people further develop their understanding. This exploratory study seeks to fill this gap through a deductive and inductive content analysis of more than 1700 definitions of the term *preprint*.

## 2. Methods

This research relies on a pragmatist interpretive framework, in which reality is understood as “what is useful, is practical, and ‘works’” (Creswell and Poth, 2017: 35) for addressing the research problem—in this case, how publics understand the term “preprint” and what factors contribute to that understanding. Given the limited prior evidence, we use research questions instead of hypotheses to drive our investigation. As is common in pragmatic research, we apply both quantitative and qualitative methods (Creswell and Poth, 2017). Specifically, we rely on deductive content analysis of open-ended data to assess how publics define preprints in ways that align with previous research (answering RQ1a) and inductive codebook thematic analysis to identify alternative definitions of preprints among participants (answering RQ1b) (Braun and Clarke, 2022). Finally, we apply descriptive and inferential statistics to examine audience characteristics and experimental manipulations of news story associated with increased preprint understanding (answering RQ2a and RQ2b).

### Sample selection

This study relies on survey responses gathered as part of a larger project examining audience responses to COVID-19 information. Responses (*N* *=* 1702) were gathered from two samples of the US general population recruited using Qualtrics Panel Services—Study 1 in March 2021 (*n* *=* 434) and Study 2 in December 2021 (*n* *=* 431)—and a student sample (Study 3) recruited from a university research pool (*n* *=* 837) in November–December 2021. The inclusion of a general population sample was important, as much of the existing research on public perceptions or knowledge of preprints has focused on student samples (e.g. Cataldo et al., 2023; Cyr et al., 2021; Wingen et al., 2022). Using both types of samples allowed us to examine whether there are potential differences in preprint understanding between students and a population with a more diverse mix of ages and education levels. All protocols were approved by the University of Utah (IRB 00131482) and University of Georgia (IRB 00003819) IRBs (Institutional Review Boards).

### Study protocol

Each of the three studies followed a similar protocol. First, participants completed a pre-exposure questionnaire that gathered demographic information. Next, participants read a modified version of a real news article describing findings of a preprint study related to COVID-19. Study 1’s article described Mardi Gras as a potential “super-spreader” event linked to increased COVID-19 cases in the state of Louisiana. The articles for Studies 2 and 3 discussed the effectiveness of “mixing and matching” different brands of COVID-19 booster vaccines.

Participants in all three studies were randomly assigned to either read a version of the news story that disclosed the study’s preprint status (e.g. labeled it a “preprint” and explained that it had not been peer reviewed or published in a scientific journal) or read a version without a preprint disclosure (i.e. with no statement that the research was a preprint or definition of the term). Although the content of these different disclosures was comparable across the three samples, the language used to disclose preprint status varied slightly (see Supplemental Material, Section 1). These variations in language—paired with the different topics of the preprint research described in each news story—enabled us to examine audiences’ understanding of preprints in diverse contexts and to explore how subtle differences in research topic or journalistic framing (i.e. news story characteristics) may have influenced their understanding, similar to Wingen et al. (2022).

After reading the news stories, participants completed attention and manipulation checks and answered a series of closed-ended questions about their perceptions of the research they had read about (the results of which are reported in Ratcliff et al. (2025) and Wicke et al. (2025)). Finally, participants answered an open-ended question: “When you see the term ‘preprint’ in a scientific news article, what do you interpret it to mean?” Responses to this question were analyzed using deductive and inductive content analysis to examine readers’ understanding of preprints, as described below.

### Measures

#### Demographics

Participants reported demographic characteristics including their age (in years) and level of education (see Table 1). We focus on age and education as potential correlates of preprint understanding, given that people with higher age or educational attainment may be more likely to have encountered preprints.

**Table 1.** Characteristics of the study samples.

Variable	Students (*n* = 837)	General population (*n* = 864)
Age	Mean = 19.19 Range: 18–29	Mean = 48.22 Range: 18–94
PIUS	Mean = 4.23 (SD = 0.50)	Mean = 3.86 (SD = 0.83)
Factual Scientific Literacy	Mean = 5.64 (SD = 1.14)	Mean = 5.03^a^ (SD = 1.40)
	*N* (%)	*N* (%)
Education		
<High school	3 (0.4)	64 (7.4)
High school/GED	269 (32.1)	369 (42.7)
Some college/associate’s degree	554 (66.2)	213 (24.7)
Four-year college degree	11 (1.3)	125 (14.5)
Advanced/professional degree	0	93 (10.8)

PIUS: preference for information about uncertain science.

The General Population sample reported here combines both subsamples.

The Student and General Population samples were significantly different on all variables *p* < .001 (see Supplemental Material, Section 6).

a Factual Scientific literacy was only measured for the Student Sample (*n* *=* 837) and for General Population Sample 1 (*n* = 433).

#### Preference for information about uncertain science

We used the 7-item Preference for Information about Uncertain Science (PIUS) scale (Ratcliff and Wicke, 2023) to assess individual preferences for learning about preliminary science and the limitations of scientific evidence, in order to examine whether having high PIUS is associated with greater awareness of the concept of a preprint. Sample scale items include “I like to learn about new scientific discoveries, even if they’re too preliminary to be acted upon” and “Science journalists should describe the uncertainties or unknowns when reporting about a scientific discovery.” Internal consistency was acceptable across datasets (Cronbach’s alpha = .74–.92). Response options ranged from 1 (*strongly disagree*) to 5 (*strongly agree*). Full items are reported in the Supplemental Material, Section 2.

#### Factual scientific literacy

We used seven statements from a common measure of factual science knowledge (National Science Board and National Science Foundation, 2020) to assess participants’ factual scientific literacy. Participants rated statements (e.g. “The center of the Earth is very hot”) as true or false. A sum score of correct answers was produced (range: 0–7). Full items are reported in the Supplemental Material, Section 2.

There were no significant differences between the two general population samples with respect to any participant characteristics or the accuracy of their preprint definitions (see Table 1 and Supplemental Material, Sections 3 and 5). As such, for simplicity, we combined them into a single general population sample (*n* = 864) for analysis, alongside the student sample (*n* = 837).

### Deductive content analysis

We first analyzed participants’ definitions of *preprint* deductively, using a codebook developed in our prior research (Ratcliff et al., 2024). This codebook was based on a definition that is commonly used among scholars in the life sciences, namely that preprints are complete scientific manuscripts that are (A) *not peer reviewed*, (B) *publicly available*, and (C) *not yet published in a scientific journal* (Berg et al., 2016). Given that preprint conclusions can change between initial posting to a preprint server and subsequent publication in a journal (Brierley et al., 2022), the codebook also included a category for indications that the evidence was (D) *preliminary or uncertain* in some way. Finally, the codebook included an (E) *other* category for definitions that did not fit into the four predefined categories, (F) a category for responses suggesting the participant *did not know* the word *preprint*, and a category for (G) *blank or irrelevant* responses. We considered definitions to be *technically accurate* if they mentioned at least one aspect of the scholarly definition of *preprint* (i.e. a definition coded as A, B, and/or C). Defining *preprint* as preliminary or uncertain evidence (code D), while not technically accurate, was considered to indicate at least a *cursory* level of understanding. The complete codebook, along with exemplar responses, is available in the Supplemental Material, Section 4.

In line with best practices for content analysis (Lacy et al., 2015), two coders (AF and CLR) independently coded a random subsample of responses to assess intercoder reliability. This subsample included 108 responses (6%) with proportional representation of each of the three study samples. This sample size has been deemed appropriate for binary coding with seven code categories and a minimum acceptable Krippendorff’s alpha of .80 (Krippendorff, 2004). Krippendorff’s alpha ranged from .88 to 1.00 for all seven categories (Table 2).

**Table 2.** Public understanding of preprint status (deductively coded responses).

Response category (ICR)		Number of instances		Example responses
		Students(*N* = 834^a^)	General population(*N* = 855^a^)	
A	Not peer reviewed (α = .91)	167 (20.02%)	41 (4.80%)	“it hasn’t been fully vetted by experts.” (1-314) “It’s an article shared before peer review” (2-261) “I think that preprint means that it has not undergone scrutiny and cannot fully be trusted yet. Preprint has not been scientifically reviewed and cannot be inferred to be 100% accurate.” (3-421)
B	Released publicly (α = .88)	29 (4.80%)	18 (2.11%)	“draft shared publicly before shared with peers.” (1-312) “something is being printed to the public before the study has been officially posted.” (3-592)
C	Not published in scientific journal (α = .88)	85 (10.19%)	40 (4.68%)	“It has not been published in a journal but is being released to show what is being done.” (1-242) “that it hasn’t been published in a medical journal yet.” (2-407)
D	Preliminary results (α = .90)	160 (19.18%)	68 (7.95%)	“It’s being talked about in print before the study is complete.” (2-169) “The article was printed before any of the data has been confirmed by science.” (3-175)
E	Other (α = .90)	429 (51.44%)	543 (63.51%)	“Easier for the reader to understand.” (1-35) “It is preliminary but generally agreed upon so almost ready to go into full printing.” (2-369) “That is not yet ready for public for fact checking reasons.” (3-40)
F	Doesn’t know (α = 1.00)	53 (6.35%)	100 (11.70%)	“I don’t know what preprint refers to.” (2-179) “Nothing I have no idea what it is for.” (3-43)
G	Irrelevant/blank (α = 1.00)	4 (0.48%)	63 (7.37%)	“na.” (2-371) “nothing.” (1-334)

Total *N* = 1689.^a^ Categories A–C are not mutually exclusive. We report Krippendorff’s alpha for ICR. The full codebook is presented in the Supplemental Material, Section 4.

Total number of responses that included at least one of A–C (i.e. technically accurate definitions): Students: 232 (27.82%), General Population: 83 (9.71%), All: 315 (18.65%). Total number of responses that included at least one of A–D (i.e. cursorily accurate definitions): Students: 359 (43.05%), General Population: 143 (16.73%), All: 502 (29.72%). Chi-square tests indicated that these responses were not likely influenced by experimental condition (see Supplemental Material, Section 5).

a From the original sample of 1702, we omitted 13 responses that were obviously copied from the Internet (10 from General Population, 3 from Students). These were identified by performing a Google search for “preprint” and “what is a preprint” and comparing the definitions provided by the first three search hits against participants’ responses. These responses were excluded from the final analyses of the open-ended data.

To further improve coding reliability, the coders then discussed discrepancies in their coding, clarified confusing or ambiguous aspects of the codebook, and refined it accordingly (cf. Krippendorff, 2004; Lacy et al., 2015). Finally, AF coded the remaining responses, seeking occasional support from CR when analyzing challenging or ambiguous responses.

### Inductive content analysis

To identify understandings of preprints not captured by our deductive coding, AF applied codebook thematic analysis to participants’ definitions of preprints. In this approach, codebooks are not used to document frequencies or achieve intercoder reliability (as with deductive content analysis) but to make coding more efficient and structured (Braun and Clarke, 2022). Codebook thematic analysis encourages ongoing researcher reflexivity, accepting the subjective lens the coder brings to analysis as an inevitable and desirable part of the research (Braun and Clarke, 2022). In this study, AF’s background in journalism, science communication, publishing, and scholarly communication—alongside her lived experiences discussing preprints with public audiences—informed the coding. For example, although codes were primarily constructed from the data, she considered research into public responses to scientific uncertainty and scholarship about academic publishing and journalism while developing themes.

The analysis was conducted in NVivo 12 for Mac and followed six phases (cf. Braun and Clarke, 2012): AF (1) familiarized herself with the data by reading through the entire dataset, (2) generated initial codes with relevance to the research problem, (3) developed preliminary themes based on patterned responses across codes and participants and drafted an initial codebook to structure her thinking, (4) critically and reflexively reviewed the themes, (5) defined and named them, and, finally, (6) refined the codebook and wove the themes together into a narrative (see Results).

### Descriptive and inferential statistics

We used Microsoft Excel to calculate the proportion of respondents who provided accurate definitions of preprints (RQ1a). To examine characteristics of the news story (RQ2a) or the participants (RQ2b) that supported accurate preprint understanding, we performed chi-square and bivariate correlation tests using SPSS v29. Correlations were all significant at *p* < .050 and were positive unless otherwise noted.

## 3. Results

### Accurate understandings of preprints

Our deductive content analysis found that almost a third of participants (*n* = 502, 29.7%; see Notes, Table 2) defined *preprint* in ways that suggested at least a cursory understanding of the term (i.e. definitions coded as A–D). Specifically, 18.7% (*n* *=* 315) of participants provided a technically accurate definition (i.e. A–C: not yet peer reviewed, publicly available, and/or has not been published in a scientific journal) and 13.5% (*n* = 228) described preprints as scientific evidence that is preliminary or uncertain (i.e. D).

However, these results mask large differences between samples, as accurate definitions of *preprint* were far more common among the student sample than among the general population samples. Among students, 27.8% (*n* = 232) defined *preprint* in technically accurate ways (A–C), whereas among the general population, only 9.7% (*n* = 83) did so. Similarly, a larger proportion of students (19.18%) defined *preprint* as preliminary or uncertain compared to the general sample (7.95%; see Table 2). In total, 43.1% (*n* = 359) of student-authored definitions suggested either a technically accurate or at least cursory understanding of preprints (i.e. A–D), whereas only 16.7% (*n* = 143) of those by general population participants did. We explore potential reasons for these differences between samples below.

### Alternative understandings of preprints

The deductive content analysis found that more than half of participants (57.6%; *n* = 972: 429 students, 543 general population) defined *preprint* in ways that did not align with any predefined coding category. The inductive content analysis shed light on these “other” definitions of preprints, which ranged from superficial descriptions of articles or information that had been “printed in advance” to deeper reflections on the trustworthiness of evidence that is preliminary, uncertain, or otherwise incomplete. Dominant themes described preprints as an aspect of the *printing and publication* process, *uncertain and incomplete information, scientific news stories*, or *complete and credible information*, as elaborated below and summarized in Supplemental Material, Section 4.

#### Printing and publication

Some participants described preprints as content “Before it has been published or will be published” (1-194) or “Something that is printed before the article actually posted” (2-352). These definitions seemed to focus primarily on the literal meaning of *pre* (i.e. before) and *print* (i.e. printed or published), with some participants admitting they were making a guess based on their understanding of these two terms. Related definitions conceptualized preprints as content that had been released in advance as a “preview” of, or an introduction to, a more complete article that would be published later. These participants offered descriptions such as, “It’s like a trailer for a movie. Gives you what’s going to come . . .” (1-357), “[Preprint] Means an introductory copy of a scientific paper . . .” (1-412), and “. . . a precursor to a larger, more expansive article regarding the same topic” (3-224). While some participants felt that these advanced publications represented content that “was printed before it was meant to be published” (3-23), others interpreted the early release as something positive. For example, one participant suggested that “maybe it was rushed out because the information was found to be extremely relevant,” (3-33) while another wondered, “Perhaps it is a print that comes before the main article as a means to get some important information out as fast as possible” (3-584).

Another group of definitions falling under the printing and publication theme described preprints as copies, reprints, or rewritten versions of content that had been printed or published previously. These participants defined a preprint as “a second printing” (1-403) or as content “reprinted by someone else to be used in a study or publication” (2-350). Interestingly, some of these descriptions suggested that plagiarism had taken place, for example, that someone “took the article from someone else” (2-390) or that “It was already written and someone copied it” (1-55). Others offered more generous interpretations, suggesting that a preprint is something that “has already been printed somewhere else and had to be re printed to correct information” (3-516). Potentially, participants may have mistaken the term *preprint* for *reprint*, given how common these types of definitions were.

Other participants defined preprints from a publishing or printing perspective by focusing on the unpublished or partially published nature of the content. Many of these descriptions emphasized that a preprint “. . . hasn’t been published yet” (1-240) or “. . . hasn’t been out in print for the public yet” (2-222), giving an interpretation opposite to the *released publicly* category from the deductive content analysis. Other participants emphasized that a preprint was “Not a fully published article” (1-92), suggesting that “. . . part of the study has been printed before the whole actual study has been officially released/printed” (3-679). These definitions were not coded as technically accurate during the deductive coding because they did not specify that the information had not been published in a *scientific journal* (i.e. category C). However, some of these alternative definitions may, in fact, have represented an accurate representation of the term *preprint* but simply failed to do so using language recognized by our deductive coding scheme.

#### Uncertain and incomplete information

Participants also described preprints as unfinished, unofficial, unverified, initial, or otherwise incomplete information. Unlike responses falling under the “uncertain evidence” category from our deductive analysis, these definitions did not describe uncertain data, research, or findings specifically; instead, they referred to more ambiguous content such as “an article,” “information,” “an idea,” or most commonly, “a draft.” Many of these responses also included a note of caution or concern, emphasizing the idea that preprints may be inaccurate, untrustworthy, likely to change, and accordingly should be taken with a grain of salt. As one student put it, “To be honest it can *make me uncertain about how effective a preprint* will be because it seems less official” (3-555, emphasis added). At the extreme, participants suggested that the information presented was suspicious and harmful, as seen in statements such as, “be car[e]ful about everything you read,” (1-415) or “. . . [I] do no[t] like nor do [I] trust that kind of science” (2-127). These cautionary comments provide some support for suggestions that audiences who are provided with a definition of preprints will see them as less credible or trustworthy than peer-reviewed research (Wingen et al., 2022).

Importantly, some participants who defined preprints this way did not see the lack of uncertainty as cause for alarm. Instead, they described the certainty or completeness of the information as distinct from its overall quality, accuracy, or trustworthiness. For example, one student said they believed *preprint* meant that, “the article is not yet ready to be published but it is on the right track to being 100% correct and ready” (3-357). While it is not possible to generalize due to the qualitative nature of the analysis, this tendency to distinguish between certainty and trustworthiness appeared to be somewhat more common among students than among the general population.

#### (Scientific) news stories

Participants also defined preprints as news stories, often offering vague descriptions such as “News about something” (1-236) or “. . . pretty new news” (2-405). Many of these definitions overlapped with the *uncertain and incomplete information* theme described above, defining preprints as “unofficial” news stories that had not yet been fully proofread, edited, fact checked, written, or approved for print. Again, some participants suggested that, as a result, the content was unconfirmed or unstable: “It is an article that is composed with basic facts that can be changed before it is made for print in national or local newspapers” (1-394). Other definitions overlapped with the *printing and publication* theme, describing preprints as information that had not yet been covered in the news or as news stories that had not yet been printed or disseminated. For example, participants offered definitions such as, “before the paper hits the newsstand for people to read it” (1-195) or “It was not printed yet onto news articles for the general public” (3-307). Again, it is possible that at least a proportion of these definitions were guesses rather than true understandings of the term *preprint*.

While some definitions describing preprints as news stories focused specifically on news production, others conflated aspects of academic and journalistic publishing. For example, participants defined *preprint* as “. . . an unofficial copy of a news article. It has not been peer reviewed” (3-311), as “A draft of the research paper or scientific news then shared with the public” (1-417), and as “. . . a study that was conducted before it was printed for people to read in the news” (2-347). In general, the terms *peer review, fact check*, and *edit* were often treated as synonyms, suggesting some confusion about what these words mean. Even in these cases—which arguably verge on accurate definitions of preprints—participants described preprints as relating to *media coverage* of science, rather than the science itself. In part, this focus on the news rather than science may be due to the phrasing of the question, which specifically asked participants to consider the term in the context of a “scientific news article.”

#### Confirmed and credible information

Finally, some responses referred to preprints as confirmed and credible sources of information. At times, these definitions reflected a clear misunderstanding of the term *preprint*, defining it as a “peer reviewed” publication or as content “. . . after the scientific peer evaluation but it still needs to be published” (3-342). Some definitions went further, explaining that the preprint status of the research made it seem more trustworthy and valid. For example, 3-807 noted that,

When I see *preprint*, I interpret it to mean that the article has been reviewed by researchers before it was published [on] the scientific news [webs]ite. This makes me trust it more since it has gone through multiple sets of eyes.

Similarly, 3-406 stated that, “When I see the term ‘preprint’ I don’t really know what that means. I would probably just assume that it [i]s a catch-all term that scientists [use] to show validity of their experiment.” Other definitions did not specify that preprints were scientific in any way but instead described the term more broadly as indicating information that “. . . has been confirmed” (1-340), is “actual fact” (2-41), or “. . . is ready and finished and ready to be printed” (2-218). As with the *uncertain and incomplete information* theme, some participants distinguished between whether information was certain or final and whether it was trustworthy, offering perspectives such as “I believe it means that the information is accurate, it just has not been officially posted yet” (3-24), or defining *preprint* as “A reliable article that has not been published yet” (2-283).

Although many definitions falling under this theme were somewhat vague, a proportion seemed to indicate that participants based their trust in the information on the quality of the science rather than the status of the publication. As one student expressed, “I honestly never heard of the word before this but as long as the study was performed in a good fashion it wouldn’t really change my opinion on the research” (3-722). This sentiment—that the quality and credibility of research is distinct from whether or not it has been peer reviewed—has been echoed by scholars who are critical of the effectiveness of peer review as a quality control mechanism (e.g. Humphries, 2021).

#### Reflections on the inductive analysis

While our qualitative approach did not allow us to draw generalizable conclusions, we noted that student participants appeared to have more nuanced or thoughtful conceptualizations of preprints overall. Regardless of whether these conceptualizations were in line with the study definition, they often showed deeper engagement with the question, through reflections on whether preprints should be trusted or by considering how they fit within their understanding of the scientific research process. In contrast, participants from the general population samples tended to provide shorter, more superficial definitions, with many focusing on the literal meaning of *preprint* as information printed or published “before” or “in advance.” However, all four themes were expressed across participants.

### Characteristics supporting preprint understanding

#### News story characteristics

To address RQ2a, we created a dichotomous variable to assess technically accurate preprint understanding; all definitions that aligned with at least one of our three predefined codes corresponding to scholarly definitions of preprints (i.e. A–C) were coded as 1 and all other responses as 0. We used chi-square tests to assess whether participants’ experimental condition (preprint-disclosure vs no-disclosure) influenced responses, allowing us to assess whether reading a news story defining the term *preprint* improves the accuracy of audience members’ understanding. As described in the “Methods” section, preprint-disclosure messages varied in the level of detail provided, with some presenting a short definition of preprint and others presenting a fuller definition. Inductively derived themes were not included in this analysis, due to their qualitative nature.

Again, we found differences between the general population and student samples. Among general population participants, there was no significant relationship between preprint disclosure and preprint understanding (χ^2^ = .13, *p* = .716). That is, participants who read a news story containing a statement that the research in question was an un-peer-reviewed preprint were no more likely to provide a technically accurate (A–C) definition of *preprint* than participants who read a news story without such a disclosure. They were also no more likely to define a preprint as uncertain evidence (i.e. D; χ^2^ = .02, *p* = .885). This was true regardless of level of detail provided in the preprint disclosures (see Supplemental material, Section 5). Among students, however, the manipulation appeared to be effective, with participants who had received the preprint disclosure being more likely to provide technically accurate definitions of *preprint* as unreviewed, not published in a journal, or publicly available (χ^2^ = 8.98, *p* = .003). Again, there was no relationship between preprint disclosures and definitions of preprint as preliminary evidence (χ^2^ = 0.15, *p* = .696).

#### Participant characteristics

Finally, addressing RQ2b, bivariate correlations revealed that several participant characteristics were associated with technically accurate definitions of preprints (see Table 3). Overall, participants who were older, more educated, had higher PIUS, and had greater factual scientific literacy were significantly more likely to provide technically accurate definitions of preprints, although all correlations were weak (all *r* < .25). Correlations were smaller for the student sample than for the general population sample. This may be because the student sample contained less variance for these variables, resulting in weaker observed relationships.

**Table 3.** Correlates of technically accurate preprint understanding.

	Students	General population
Age	.07*	.13**
Education	–	.18**
PIUS	.08*	.18**
Factual Scientific Literacy	.14*	.23**^a^

PIUS: preference for information about uncertain science.

Technically accurate preprint understanding based on definitions corresponding to codes A–C = not peer reviewed, publicly available, and/or not published in a journal.

Correlates of defining preprint as preliminary (code D) are reported in the Supplemental Material, Section 7. A small correlation emerged between PIUS and defining preprint as preliminary in the general population; otherwise, no relationships were significant.

a Correlation for Factual Scientific Literacy based only on data from the Student Sample and General Population Sample in Study 1, as it was not measured for the General Population Sample in Study 2. No correlation was reported for students’ education because all students had the same numeric value for education as measured.

* *p* < .05; **p < .01.

## 4. Discussion

Preprints use has been increasing in scholarly communication (Xie et al., 2021) and journalism (Fleerackers et al., 2024b), yet little is known about whether or how nonexperts understand preprints and how they differ from peer-reviewed studies (Berenbaum, 2023; Gopalakrishna, 2021). Through an analysis of more than 1700 definitions of the term *preprint* generated by US citizens and students, this study contributes to filling this gap by shedding light on the many ways in which publics understand (or misunderstand) the term *preprint* when it is mentioned in a news story, and what characteristics of the audience and the news coverage support more accurate understanding. We found that only about one in five people were able to define *preprint* in ways that align with scholarly conceptualizations of the term, similar to our previous research (Ratcliff et al., 2024). However, unlike in previous studies, we also captured a rich array of “other” conceptualizations of preprints, some of which suggested at least a partial understanding of the term. For example, participants described preprints as incomplete or uncertain information, rough “drafts,” or content that was complete but had not yet been officially published, but did not specify that the content was research. Other definitions, however, indicated considerable confusion among participants—such as those that equated preprints to plagiarized or reprinted content, un-proofread versions of news stories, or settled science. Collectively, these results support growing concerns that publics do not understand the term *preprint* (Fleerackers et al., 2022a; Kardos et al., 2023; Simons and Schniedermann, 2023). This lack of understanding poses a challenge for how to communicate about these unreviewed studies in ways that will resonate with nonscientific audiences.

With respect to this challenge, we found that providing a brief definition of the term *preprint* in a news story only led to improved preprint understanding among students; for other US citizens, explaining that preprints are unreviewed research papers that are publicly available but have not been published in a journal made no significant difference in their ability to define the term accurately. Relatedly, participants who were older, more educated, and had higher scientific literacy were more likely to provide definitions of *preprint* that align with how scholars understand the term (Berg et al., 2016). These findings suggest that guidelines which recommend labeling these studies as unreviewed preprints (e.g. ASAPbio, n.d.) may not be effective for helping less educated or scientifically literate members of the public evaluate or make sense of the research. Although the conclusions of many preprints remain largely unchanged between posting to a preprint server and peer-reviewed publication (Brierley et al., 2022), in those instances where findings change considerably, failure to communicate about preprints in ways that less educated audiences can understand could contribute to the spread of confusion, doubt, and, potentially, misinformation (Chiarelli et al., 2019; Fleerackers et al., 2022a).

At the same time, our findings provide a grounding to develop strategies for reporting on preprints that may be more intuitive to audiences. For example, our results suggest a tendency among both students and members of the general population to conceptualize preprints as “unofficial drafts,” “previews,” or “trailers.” Future studies could examine whether using similar, but more accurate, versions of these metaphors to explain preprint status can better support audiences in making sense of the research. Similarly, our study revealed several common misinterpretations of the term *preprint*—for example, as something yet to be printed or published, incomplete research, or un-proofread news. Educators, journalists, and science communicators could support improved public understanding of preprints by addressing these common misconceptions and exploring new ways of communicating about preprints that avoid encouraging such framings.

Finally, the results of the qualitative analysis suggest that, at least for some members of the public, understanding preprints as preliminary, unfinished, or draft content does not undermine their credibility. Instead, for these individuals, preprints appear to represent both unfinalized science and valid, trustworthy information—a perspective that is shared by some scholars (Vianello, 2021) and is supported by research documenting the limited changes many preprints undergo during peer review (Brierley, 2021; Nicholson et al., 2022). Although these individuals represented a minority in our study sample, Ratcliff et al. (2024) similarly found that reading a news story describing research as an unreviewed preprint did not undermine audience members’ trust in either the news story or the scientists behind the research. Only when the news story framed the research as uncertain, with explicit discussion of knowledge gaps, did the audience lose trust. Collectively, these findings could suggest that, at least for some individuals, communication that is transparent about a preprint’s status does not automatically undermine the credibility of the research or the news story. This could have positive implications for journalists, as being transparent and accurate about uncertainties is a core principle of ethical science and risk communication (Covello and Allen, 1988; Medvecky and Leach, 2019) but communicators sometimes worry that conveying uncertainties will generate negative public responses.

### Limitations and future directions

These findings must be understood in light of certain limitations. First, participants were all based in the US, which leaves much unknown about how publics in other countries may understand preprints or respond to disclosures of preprint status they encounter in the news. In addition, it is possible that some of the definitions of *preprint* that participants provided—especially those that equated preprints to documents before they are printed or published—were best guesses rather than a true conceptualization of the term. As such, some of these definitions may not reflect alternative understandings of preprints but a lack of knowledge of what they are. It is also worth noting that many of the definitions provided by participants were very close to those outlined in our predetermined (deductive) categories but failed to meet all of our coding criteria. For example, many participants referred to studies that had not been “officially published” (but did not mention journals or academic publishing) or that were not “verified” (but did not mention peers or experts). This could suggest that many participants had some understanding of preprints but did not articulate it in ways that aligned with our codebook; as such, the results of the deductive analysis may underestimate the proportion of audience members who understand the term *preprint*. In addition, this research was conducted in the second year of the COVID-19 pandemic and used stimuli that related to controversial and politicized topics (e.g. COVID-19 vaccinations, see Khubchandani et al., 2021). Given that public responses to uncertainty related to controversial science—especially COVID-19 science—can differ from responses to other forms of uncertainty (Gustafson and Rice, 2019; Ratcliff et al., 2022), it is possible that research conducted at different times, or related to different science topics, would yield different results.

More broadly, our results raise several questions that warrant future research. First, it is unclear why people with greater PIUS tended to provide more technically accurate definitions of *preprint* than others. Given that this measure captures both a preference to receive information about scientific uncertainties and caveats, and a preference to learn about science when it is still in a preliminary state (Ratcliff and Wicke, 2023), it is possible that high-PIUS individuals had consumed more news (or other forms of science communication) about preliminary science before participating in the study, some of which may have included explanations of the term *preprint*. Alternatively, they may have been more sensitive to language indicating uncertainty, such as statements that research is not yet peer reviewed, similar to those included in the stimuli for the experimental conditions in this study. Overall, high-PIUS individuals tend to exhibit a stronger understanding of science (Ratcliff et al., 2023). More research is needed to better understand the connection between preprint understanding and PIUS, as well as other personal characteristics, such as level of science news exposure or interest in science.

Relatedly, it is unclear why students were more adept at defining *preprint* than members of the general population. It is likely that education played a role (as students had a higher average level of education and, perhaps relatedly, factual scientific literacy than the general population sample). However, other elements may also have come into play, such as different levels of understanding of the internal processes of science (e.g. the nature of peer review, scientific uncertainty, journal publishing). Indeed, our measure of scientific literacy focused solely on participants’ understanding of specific natural science facts, rather than knowledge of how science works. Future research could examine whether such scientific process knowledge supports improved public understanding of preprints, in line with arguments put forward by Millar and Wynne (1988).

Finally, although we found that providing a definition of the term *preprint* in a news story did not help most participants correctly define this term, it is possible that such definitions affected other outcomes, such as participants’ understanding of the uncertainty related to the findings. More research is needed to assess whether news stories discussing preprints or the peer review process impact public perceptions or understanding of research in ways not measured in this study, especially in the long term. Addressing these and related questions could help shed light on the cognitive processes that underpin individuals’ responses to preprint definitions and ultimately provide a theoretical grounding for this emerging but undertheorized area of scholarship.

## 5. Conclusion

Preprints use has been increasing in scholarly communication, science communication, and journalism. These unreviewed studies have the potential to provide evidence-based insights that could be used to address societal challenges in a timelier fashion than is possible with peer-reviewed research. At the same time, they could lead to confusion, misinformation, or poorly informed decision making among the public if the findings do not hold up during peer review. As such, developing strategies for communicating about the unreviewed nature of preprints in ways that nonexperts can understand is essential. This study provides initial evidence into how publics understand—or, more often, *misunderstand*—the term, paving the way for the development of such strategies and, ultimately, for more transparent and ethical science communication.

## Supplemental Material

sj-docx-1-pus-10.1177_09636625241268881 – Supplemental material for Public understanding of preprints: How audiences make sense of unreviewed research in the news Supplemental material, sj-docx-1-pus-10.1177_09636625241268881 for Public understanding of preprints: How audiences make sense of unreviewed research in the news by Alice Fleerackers, Chelsea L. Ratcliff, Rebekah Wicke, Andy J. King and Jakob D. Jensen in Public Understanding of Science

## References

[bibr1-09636625241268881] ASAPbio (n.d.) Guiding principles and resources for journalists and science writers to aid the responsible media reporting of research posted as preprints. Available at: https://asapbio.org/wp-content/uploads/2021/03/Preprints-in-the-Public-Eye-Journalists.pdf

[bibr2-09636625241268881] Berenbaum MR (2023) On peer review—Then, now, and soon to be? Proceedings of the National Academy of Sciences 120(11): e2302593120. 10.1073/pnas.2302593120 PMC10242717 36888652

[bibr3-09636625241268881] Berg JM Bhalla N Bourne PE Chalfie M Drubin DG Fraser JS , et al. (2016) Preprints for the life sciences. Science 352(6288): 899–901. 27199406 10.1126/science.aaf9133

[bibr4-09636625241268881] Braun V Clarke V (2012) Thematic analysis. In: Cooper H Camic PM Long DL , et al. (eds) APA Handbook of Research Methods in Psychology, Vol 2: Research Designs: Quantitative, Qualitative, Neuropsychological, and Biological. Washington, DC: American Psychological Association, pp. 57–71.

[bibr5-09636625241268881] Braun V Clarke V (2022) Conceptual and design thinking for thematic analysis. Qualitative Psychology 9(1): 3–26.

[bibr6-09636625241268881] Brierley L (2021) Lessons from the influx of preprints during the early COVID-19 pandemic. The Lancet Planetary Health 5(3): e115–e117. 33713612 10.1016/S2542-5196(21)00011-5

[bibr7-09636625241268881] Brierley L Nanni F Polka JK Dey G Pálfy M Fraser N , et al. (2022) Tracking changes between preprint posting and journal publication during a pandemic. PLoS Biology 20(2): e3001285. 10.1371/journal.pbio.3001285 PMC8806067 35104285

[bibr8-09636625241268881] Cataldo T Faniel I Buhler A Brannon B Connaway LS Putnam S (2023) Students’ perceptions of preprints discovered in Google: A window into recognition and evaluation. College & Research Libraries 84(1): 137–153.

[bibr9-09636625241268881] Caulfield T Bubela T Kimmelman J Ravitsky V (2021) Let’s do better: Public representations of COVID-19 science. Facets 6(1): 403–423.

[bibr10-09636625241268881] Chiarelli A Johnson R Pinfield S Richens E (2019) Preprints and scholarly communication: An exploratory qualitative study of adoption, practices, drivers and barriers [version 2; peer review: 3 approved, 1 approved with reservations]. F1000Research 8: 971. 32055396 10.12688/f1000research.19619.2 PMC6961415

[bibr11-09636625241268881] Covello VT Allen F (1988) Seven Cardinal Rules of Risk Communication. Washington, DC: US Environmental Protection Agency, Office of Policy Analysis.

[bibr12-09636625241268881] Creswell J Poth C (2017) Qualitative Inquiry and Research Design: Choosing among Five Approaches, 4th edn. Los Angeles, CA: Sage.

[bibr13-09636625241268881] Cyr C Cataldo TT Brannon B Buhler AG Faniel IM Connaway LS , et al. (2021) Backgrounds and behaviors: Which students successfully identify online resources in the face of container collapse. First Monday 26: 10871.

[bibr14-09636625241268881] Drzymalla E Yu W Khoury MJ Gwinn M . (2022) COVID-19-related manuscripts: Lag from preprint to publication. BMC Research Notes 15(1): Article 340. 10.1186/s13104-022-06231-9 PMC9636814 36335379

[bibr15-09636625241268881] Fleerackers A Chtena N Pinfield S Alperin JP Barata G Oliveira M , et al. (2024a) Making science public: A review of journalists’ use of Open Access research [version 2; peer review: 5 approved]. F1000Research 12: 512. 37920454 10.12688/f1000research.133710.2 PMC10618641

[bibr16-09636625241268881] Fleerackers A Moorhead LL Maggio LA Fagan K Alperin JP (2022a) Science in motion: A qualitative analysis of journalists’ use and perception of preprints. PLoS ONE 17(11): e0277769. 10.1371/journal.pone.0277769 PMC9678308 36409723

[bibr17-09636625241268881] Fleerackers A Riedlinger M Moorhead LL Ahmed R Alperin JP (2022b) Communicating scientific uncertainty in an age of COVID-19: An investigation into the use of preprints by digital media outlets. Health Communication 37(6): 726–738. 33390033 10.1080/10410236.2020.1864892

[bibr18-09636625241268881] Fleerackers A Shores K Chtena N Alperin JP (2024b) Unreviewed science in the news: The evolution of preprint media coverage from 2014–2021. Quantitative Science Studies 5(2): 297–316.

[bibr19-09636625241268881] Fraser J Polka J (2018) Preprints: Safeguard rigour together. Nature 560: 553. 10.1038/d41586-018-06053-5 30158617

[bibr20-09636625241268881] Fraser N Brierley L Dey G Polka JK Pálfy M Nanni F , et al. (2021) The evolving role of preprints in the dissemination of COVID-19 research and their impact on the science communication landscape. PLoS Biology 19(4): e3000959. 10.1371/journal.pbio.3000959 PMC8046348 33798194

[bibr21-09636625241268881] Froke P Bratton AJ McMillan J Sarkar P Schwartz J Vadarevu R (2020) Health, science and environment reporting. In: Froke P Bratton AJ McMillan J , et al. (eds) The Associated Press Stylebook 2020–2022, 55th edn. New York, NY: Basic Books.

[bibr22-09636625241268881] Ginsparg P (2021) Lessons from arXiv’s 30 years of information sharing. Nature Reviews Physics 3(9): 602–603. 10.1038/s42254-021-00360-z PMC8335983 34377944

[bibr23-09636625241268881] Gopalakrishna G (2021) Preprint advocates must also fight for research integrity. Nature. Epub ahead of print 13 September. DOI: 10.1038/d41586-021-02481-y. 34518692

[bibr24-09636625241268881] Gustafson A Rice RE (2019) The effects of uncertainty frames in three science communication topics. Science Communication 41(6): 679–706.

[bibr25-09636625241268881] Humphries M (2021) The absurdity of peer review. Elemental, 3 June. Available at: https://elemental.medium.com/the-absurdity-of-peer-review-1d58e5d9e661/ (accessed 23 June 2021).

[bibr26-09636625241268881] Johansson MA Reich NG Meyers LA Lipsitch M (2018) Preprints: An underutilized mechanism to accelerate outbreak science. PLoS Medicine 15(4): e1002549. 10.1371/journal.pmed.1002549 PMC5882117 29614073

[bibr27-09636625241268881] Kardos P Kun Pléh ÁC Jordán F (2023) (How) should researchers publicize their research papers before peer review? Scientometrics 128: 2019–2013. 10.1007/s11192-023-04646-0 PMC9896438 36777380

[bibr28-09636625241268881] Khubchandani J Sharma S Price JH Wiblishauser MJ Sharma M Webb FJ (2021) COVID-19 vaccination hesitancy in the United States: A rapid national assessment. Journal of Community Health 46(2): 270–277. 33389421 10.1007/s10900-020-00958-x PMC7778842

[bibr29-09636625241268881] Krippendorff K (2004) Content Analysis: An Introduction to Its Methodology, 2nd edn. Thousand Oaks, CA: Sage.

[bibr30-09636625241268881] Lacy S Watson BR Riffe D Lovejoy J (2015) Issues and best practices in content analysis. Journalism & Mass Communication Quarterly 92(4): 791–811.

[bibr31-09636625241268881] Majumder MS Mandl KD (2020) Early in the epidemic: Impact of preprints on global discourse about COVID-19 transmissibility. The Lancet Global Health 8(5): e627–e630. 10.1016/S2214-109X(20)30113-3 PMC7159059 32220289

[bibr32-09636625241268881] Massarani L Neves LFF (2022) Reporting COVID-19 preprints: Fast science in newspapers in the United States, the United Kingdom and Brazil. Ciência & Saúde Coletiva 27: 957–968. 35293473 10.1590/1413-81232022273.20512021

[bibr33-09636625241268881] Medvecky F Leach J (2019) An Ethics of Science Communication. London and Cham: Palgrave Pivot.

[bibr34-09636625241268881] Millar R Wynne B (1988) Public understanding of science: From contents to processes. International Journal of Science Education 10(4): 388–398.

[bibr35-09636625241268881] National Science Board and National Science Foundation (2020) Science and Technology: Public Attitudes, Knowledge, and Interest. Alexandria, VA: Science and Engineering Indicators. Available at: https://ncses.nsf.gov/pubs/nsb20207/

[bibr36-09636625241268881] Nicholson DN Rubinetti V Hu D Thielk M Hunter LE Greene CS (2022) Examining linguistic shifts between preprints and publications. PLoS Biology 20(2): e3001470. 10.1371/journal.pbio.3001470 PMC8806061 35104289

[bibr37-09636625241268881] Oliveira T Araujo RF Cerqueira RC Pedri P (2021) Politização de controvérsias científicas pela mídia brasileira em tempos de pandemia: a circulação de preprints sobre Covid-19 e seus reflexos [Politicization of scientific controversies by the Brazilian media in times of pandemic: the circulation of preprints about Covid-19 and its reflections]. Revista Brasileira de História da Mídia 10(1): 30–52.

[bibr38-09636625241268881] Puebla I Polka J Rieger OY (2022) Preprints: Their Evolving Role in Science Communication. Mountain View, CA: Against the Grain.

[bibr39-09636625241268881] Ratcliff CL Fleerackers A Wicke R Harvilld B King AJ Jensen JD (2024) Framing COVID-19 preprint research as uncertain: A mixed-method study of public reactions. Health Communication 39(2): 283–296. 36683347 10.1080/10410236.2023.2164954

[bibr40-09636625241268881] Ratcliff CL Fleerackers A Wicke R King AJ Jensen JD (2025). Do news audiences appreciate details about a study’s preprint status? Message perceptions and amount of detail matter. SocArXiv [preprint]. Available at: 10.31235/osf.io/4u2z7

[bibr41-09636625241268881] Ratcliff CL Harvill B Wicke R (2023) Understanding public preferences for learning about uncertain science: Measurement and individual difference correlates. Frontiers in Communication 8: 1245786.

[bibr42-09636625241268881] Ratcliff CL Wicke R Harvill B (2022) Communicating uncertainty to the public during the COVID-19 pandemic: A scoping review of the literature. Annals of the International Communication Association 46(4): 260–289.

[bibr43-09636625241268881] Ratcliff CL Wicke R (2023) How the public evaluates media representations of uncertain science: An integrated explanatory framework. Public Understanding of Science 32(4): 410–427. 36196654 10.1177/09636625221122960

[bibr44-09636625241268881] Sarabipour S (2018) Preprints: Good for science and public. Nature 560: 553. 10.1038/d41586-018-06054-4 30158619

[bibr45-09636625241268881] Schultz T (2023) A survey of US science journalists’ knowledge and opinions of open access research. International Journal of Communication 17: 2732–2753.

[bibr46-09636625241268881] Sense About Science (2009) Peer review survey 2009: Full report. Available at: https://senseaboutscience.org/wp-content/uploads/2016/12/Peer_Review_Survey.pdf

[bibr47-09636625241268881] Sheldon T (2018a) Preprints could promote confusion and distortion. Nature 559(7715): 445. 30042547 10.1038/d41586-018-05789-4

[bibr48-09636625241268881] Sheldon T (2018b) The impact of preprint on media reporting of science. The Lancet 392(10154): 1194. 10.1016/S0140-6736(18)31871-3 30319106

[bibr49-09636625241268881] Simons A Schniedermann A (2023) Preprints in the German news media before and during the COVID pandemic: A comparative mixed-method analysis. In: Broer I Lemke S Mazarakis A , et al. (eds) The Science-Media-Interface: On the Relation between Internal and External Science Communication. Berlin: De Gruyter Saur, pp. 53–78.

[bibr50-09636625241268881] Song H Markowitz DM Taylor SH (2022) Trusting on the shoulders of open giants? Open science increases trust in science for the public and academics. Journal of Communication 72(4): 497–510.

[bibr51-09636625241268881] Van Schalkwyk F Dudek J (2022) Reporting preprints in the media during the Covid-19 pandemic. Public Understanding of Science 31(5): 608–616. 35196912 10.1177/09636625221077392 PMC9160779

[bibr52-09636625241268881] Van Schalkwyk MCI Hird TR Maani N Petticrew M Gilmore AB (2020) The perils of preprints. BMJ 370: m3111. 10.1136/bmj.m3111 32816814

[bibr53-09636625241268881] Vianello SD (2021) The “pre” in [my] “preprint” is for pre-figurative. Commonplace. Epub ahead of print 7 October. DOI: 10.21428/6ffd8432.5de25622.

[bibr55-09636625241268881] Wicke R Ratcliff CL Fleerackers A Wang Y King AJ Jensen JD (2025). Preprints in COVID-19 news coverage: Comparing student and general population perceptions of preliminary science about booster vaccination. SocArXiv [preprint]. Available at: 10.31219/osf.io/g3rm9 40583032

[bibr56-09636625241268881] Wingen T Berkessel JB Dohle S (2022) Caution, preprint! Brief explanations allow nonscientists to differentiate between preprints and peer-reviewed journal articles. Advances in Methods and Practices in Psychological Science 5(1): 25152459211070559.

[bibr57-09636625241268881] Xie B Shen Z Wang K (2021) Is preprint the future of science? A thirty year journey of online preprint services. arXiv [preprint]. DOI: 10.48550/arXiv.2102.09066.

