# Supplementary material for: Public understanding of preprints: How audiences make sense of unreviewed research in the news
Source: Public Underst Sci. 2024 Oct 11;34(2):154–71. doi: 10.1177/09636625241268881 (PMC11783973; doi:10.1177/09636625241268881)
Supplement: sj-docx-1-pus-10.1177_09636625241268881 – Supplemental material for Public understanding of preprints: How audiences make sense of unreviewed research in the news [file sj-docx-1-pus-10.1177_09636625241268881.docx]

**Supplemental Material**

Supplement to the article:

**Public understanding of preprints: How audiences make sense
of unreviewed research in the news**

Alice Fleerackers^1,2^, Chelsea L. Ratcliff^3^, Rebekah Wicke^4^,
Andy J. King^5^, and Jakob D. Jensen^5^

^1^Journalism, Writing, and Media, University of British Columbia, Vancouver, BC, Canada, ^2^Interdisciplinary Studies, Simon Fraser University, Vancouver, BC, Canada, ^3^Communication Studies, University of Georgia, Athens, GA, USA, ^4^Communication, Cornell University, Ithaca, NY, USA, ^5^Communication and Huntsman Cancer Institute, University of Utah, Salt Lake City, UT, USA

**Contents**

SM 1. News Story Characteristics (Experimental Message Text)

SM 2. Audience Characteristic Measures

SM 3. Participant Characteristics by Sample

SM 4. Codebook and Sample Responses for Deductive and Inductive Content Analysis of Open-ended Data

SM 5. Preprint Definitions by Experimental Message Version

SM 6. Differences in Preprint Understanding and Individual Characteristics Between Student and General Population Samples

SM 7. Individual Difference Correlates of Defining Preprint as Preliminary

**Supplemental Material 1**

***News Story Characteristics (Experimental Manipulations)***

| **Condition** | **Manipulations** | |
| --- | --- | --- |
|  | *Sample 1 (General Population): Mardi Gras as a super spreader event* | *Sample 2 (Students) and 3 (General Populations): Mixing and matching booster vaccines* |
| Control | “A new study”  “The research”  “This data” | “A recent study”  “Data from the study”  “The study”  “these study results” |
| Preprint  (brief disclosure) | “A new preprint study”  “The research, which has yet to go through scientific peer review”  “This data” | N/A |
| Preprint (expanded disclosure) | “A new preprint released by the authors”  “The research, which has yet to go through scientific peer review”  “This data, which has not yet been published in a scientific journal” | “A recent preprint study, not yet evaluated by outside experts or published in a scientific journal”  “Data from the preprint study”  “The preprint research”  “these preprint study results” |
| Notes. For samples 2 and 3, the stimuli varied in whether scientific claims were hedged or unhedged. See the full experimental message text for each sample below. | | |

***Full Experimental Messages with Manipulations Highlighted***

*Sample 1 (General Population): Mardi Gras as a super spreader event*

| **Control condition** |
| --- |
| **Can Louisiana’s COVID surge trace back to one Mardi Gras reveler?**  ***A new study suggests that last year’s Mardi Gras celebrations were responsible for roughly 50,000 COVID-19 cases across the state.***  Mardi Gras, held on February 25 of last year, was one of New Orleans’ last normal moments before the pandemic. COVID-19 was already confirmed in the Seattle area, but the federal government had yet to warn other states of the danger.  Robert Garry, a virologist who studies emerging diseases at Tulane University, remembers waiting for his daughters to roll past in a parade, and as he watched the crowd gather, thinking, “yea, [COVID] could be at an event like that.”  But at the time, he says, given the lack of national guidance and diagnosed cases, “I don’t think it would have been appropriate to stand up and say, hey, we’ve got to cancel Mardi Gras now.”  A new study, released on Medrxiv, offers one of the first hard analyses showing that, yes, a single individual may have kicked off a chain of transmission during the holiday, eventually leading to 50,000 infections in Louisiana.  The research uses several lines of evidence to reconstruct when the virus first arrived in the city, how quickly it spread, and where it ended up over the following months. And it highlights the extent to which bad luck and individual transmission events have shaped the course of the pandemic across the country.  “Mardi Gras was probably an ideal situation for a virus like this to spread,” says Garry, who was a senior author on the paper. “A lot of people getting together for Mardi Gras balls, getting together in close spaces, eating and drinking and chatting.”  This data comes to light just as Mardi Gras comes around again. The holiday is on February 16, but the usual traditions have  been put into a kind of limbo. The researchers say that in light of their findings, it’s a wise decision, and one that should be taken to heart nationally. “You cannot have big events like Mardi Gras when you’re trying to control the spread of a virus,” Gangavarapu says. “You don’t know how many cases it’s going to cause.” |
| **Preprint brief disclosure** |
| **Can Louisiana’s COVID surge trace back to one Mardi Gras reveler?**  ***A new preprint study suggests that last year’s Mardi Gras celebrations were responsible for roughly 50,000 COVID-19 cases across the state.***  Mardi Gras, held on February 25 of last year, was one of New Orleans’ last normal moments before the pandemic. COVID-19 was already confirmed in the Seattle area, but the federal government had yet to warn other states of the danger.  Robert Garry, a virologist who studies emerging diseases at Tulane University, remembers waiting for his daughters to roll past in a parade, and as he watched the crowd gather, thinking, “yea, [COVID] could be at an event like that.”  But at the time, he says, given the lack of national guidance and diagnosed cases, “I don’t think it would have been appropriate to stand up and say, hey, we’ve got to cancel Mardi Gras now.”  A new preprint, released on the research platform Medrxiv, offers one of the first hard analyses showing that, yes, a single individual may have kicked off a chain of transmission during the holiday, eventually leading to 50,000 infections in Louisiana.  The research, which has yet to go through scientific peer review, uses several lines of evidence to reconstruct when the virus first arrived in the city, how quickly it spread, and where it ended up over the following months. And it highlights the extent to which bad luck and individual transmission events have shaped the course of the pandemic across the country.  “Mardi Gras was probably an ideal situation for a virus like this to spread,” says Garry, who was a senior author on the paper. “A lot of people getting together for Mardi Gras balls, getting together in close spaces, eating and drinking and chatting.”  This data comes to light just as Mardi Gras comes around again. The holiday is on February 16, but the usual traditions have  been put into a kind of limbo. The researchers say that in light of their findings, it’s a wise decision, and one that should be taken to heart nationally. “You cannot have big events like Mardi Gras when you’re trying to control the spread of a virus,” Gangavarapu says. “You don’t know how many cases it’s going to cause.” |
| **Preprint expanded disclosure** |
| **Can Louisiana’s COVID surge trace back to one Mardi Gras reveler?**  ***A new preprint study suggests that last year’s Mardi Gras celebrations were responsible for roughly 50,000 COVID-19 cases across the state.***  Mardi Gras, held on February 25 of last year, was one of New Orleans’ last normal moments before the pandemic. COVID-19 was already confirmed in the Seattle area, but the federal government had yet to warn other states of the danger.  Robert Garry, a virologist who studies emerging diseases at Tulane University, remembers waiting for his daughters to roll past in a parade, and as he watched the crowd gather, thinking, “yea, [COVID] could be at an event like that.”  But at the time, he says, given the lack of national guidance and diagnosed cases, “I don’t think it would have been appropriate to stand up and say, hey, we’ve got to cancel Mardi Gras now.”  A new preprint, released by the authors on the research platform Medrxiv, offers one of the first hard analyses showing that, yes, a single individual may have kicked off a chain of transmission during the holiday, eventually leading to 50,000 infections in Louisiana.  The research, which has yet to go through scientific peer review, uses several lines of evidence to reconstruct when the virus first arrived in the city, how quickly it spread, and where it ended up over the following months. And it highlights the extent to which bad luck and individual transmission events have shaped the course of the pandemic across the country.  “Mardi Gras was probably an ideal situation for a virus like this to spread,” says Garry, who was a senior author on the paper. “A lot of people getting together for Mardi Gras balls, getting together in close spaces, eating and drinking and chatting.”  This data, which has not yet been published in a scientific journal, comes to light just as Mardi Gras comes around again. The holiday is on February 16, but the usual traditions have  been put into a kind of limbo. The researchers say that in light of their findings, it’s a wise decision, and one that should be taken to heart nationally. “You cannot have big events like Mardi Gras when you’re trying to control the spread of a virus,” Gangavarapu says. “You don’t know how many cases it’s going to cause.” |

*Sample 2 (Students) and 3 (General Populations): Mixing and matching booster vaccines*

| **Control condition – hedged scientific claims** |
| --- |
| **FDA to allow ‘mix-and-match’ approach on coronavirus booster vaccines**  ***Study suggests people may bolster protection by getting an extra shot, even if it is from***  ***another brand***  The Food and Drug Administration said this week that people can get coronavirus vaccine booster shots that are different from their initial doses.  The agency made the announcement Wednesday as part of its authorization of boosters for the Moderna and Johnson & Johnson vaccines.  Johnson & Johnson’s vaccine has been shown to be less effective than the Pfizer-BioNTech and Moderna vaccines, so there is intense focus on how to boost protection for recipients of the single-shot Johnson & Johnson regimen: Should it be with the Johnson & Johnson shot or with the other vaccines, which use a different technology?  “People should generally get the same vaccine as their initial series,” said a federal official. “But some people might not have access to their original vaccine, and some people might have had a bad reaction to the mRNA vaccines — the technology used to make the Pfizer-BioNTech and Moderna shots.”  A recent study that tested mixing booster doses from different companies may provide answers. The findings could be good news for the 15 million people who received the Johnson & Johnson vaccine in the United States, many of whom have felt left out because the vast majority of U.S. vaccine recipients received either the Pfizer-BioNTech or Moderna vaccine.  Data from the study suggested that while recipients of the Johnson & Johnson coronavirus vaccine can benefit from a second dose of the original, they may derive even greater protection if the boost comes from a different vaccine technology.  The study showed that a second shot using the Moderna vaccine triggered the biggest boost of virus-neutralizing antibodies in Johnson & Johnson recipients, resulting in a 76-fold increase in antibody levels. A Pfizer booster increased antibody levels 35-fold. A matching Johnson & Johnson booster triggered only a 4-fold increase.  The take-home message from these study results is that those who need to “mix and match” their booster shot and original vaccine – especially if the original was Johnson & Johnson – could still receive a protective benefit. |
| **Control condition – unhedged scientific claims** |
| **FDA to allow ‘mix-and-match’ approach on coronavirus booster vaccines**  ***Study shows people can bolster protection by getting an extra shot, even if it is from another brand***  The Food and Drug Administration said this week that people can get coronavirus vaccine booster shots that are different from their initial doses.  The agency made the announcement Wednesday as part of its authorization of boosters for the Moderna and Johnson & Johnson vaccines.  “People should generally get the same vaccine as their initial series,” said a federal official. “But some people might not have access to their original vaccine, and some people might have had a bad reaction to the mRNA vaccines — the technology used to make the Pfizer-BioNTech and Moderna shots.” |
| A recent study that tested mixing booster doses from different companies provides answers. The findings are good news for the 15 million people who received the Johnson & Johnson vaccine in the United States, many of whom have felt left out because the vast majority of U.S. vaccine recipients received either the Pfizer-BioNTech or Moderna vaccine.  Data from the study showed that while recipients of the Johnson & Johnson coronavirus vaccine can benefit from a second dose of the original, they may derive even greater protection if the boost comes from a different vaccine technology.  The study showed that a second shot using the Moderna vaccine triggered the biggest boost of virus-neutralizing antibodies in Johnson & Johnson recipients, resulting in a 76-fold increase in antibody levels. A Pfizer booster increased antibody levels 35-fold. A matching Johnson & Johnson booster triggered only a 4-fold increase.  The take-home message from these study results is that those who need to “mix and match” their booster shot and original vaccine – especially if the original was Johnson & Johnson – will still receive a protective benefit. |
| **Preprint expanded disclosure – hedged scientific claims** |
| **FDA to allow ‘mix-and-match’ approach on coronavirus booster vaccines**  ***Preprint study suggests people may bolster protection by getting an extra shot, even if it is from another brand***  The Food and Drug Administration said this week that people can get coronavirus vaccine booster shots that are different from their initial doses.  The agency made the announcement Wednesday as part of its authorization of boosters for the Moderna and Johnson & Johnson vaccines.  Johnson & Johnson’s vaccine has been shown to be less effective than the Pfizer-BioNTech and Moderna vaccines, so there is intense focus on how to boost protection for recipients of the single-shot Johnson & Johnson regimen: Should it be with the Johnson & Johnson shot or with the other vaccines, which use a different technology?  “People should generally get the same vaccine as their initial series,” said a federal official. “But some people might not have access to their original vaccine, and some people might have had a bad reaction to the mRNA vaccines — the technology used to make the Pfizer-BioNTech and Moderna shots.”  A recent preprint study, not yet evaluated by outside experts or published in a scientific journal, may provide answers. The study tested mixing booster doses from different companies The findings could be good news for the 15 million people who received the Johnson & Johnson vaccine in the United States, many of whom have felt left out because the vast majority of U.S. vaccine recipients received either the Pfizer-BioNTech or Moderna vaccine.  Data from the preprint study suggested that while recipients of the Johnson & Johnson coronavirus vaccine can benefit from a second dose of the original, they may derive even greater protection if the boost comes from a different vaccine technology.  The preprint research showed that a second shot using the Moderna vaccine triggered the biggest boost of virus-neutralizing antibodies in Johnson & Johnson recipients, resulting in a 76-fold increase in antibody levels. A Pfizer booster increased antibody levels 35-fold. A matching Johnson & Johnson booster triggered only a 4-fold increase.  The take-home message from these preprint study results is that those who need to “mix and match” their booster shot and original vaccine – especially if the original was Johnson & Johnson – could still receive a protective benefit. |
| **Preprint expanded disclosure – unhedged scientific claims** |
| **FDA to allow ‘mix-and-match’ approach on coronavirus booster vaccines**  ***Preprint study shows people can bolster protection by getting an extra shot, even if it is from another brand***  The Food and Drug Administration said this week that people can get coronavirus vaccine booster shots that are different from their initial doses.  The agency made the announcement Wednesday as part of its authorization of boosters for the Moderna and Johnson & Johnson vaccines.  Johnson & Johnson’s vaccine has been shown to be less effective than the Pfizer-BioNTech and Moderna vaccines, so there is intense focus on how to boost protection for recipients of the single-shot Johnson & Johnson regimen: Should it be with the Johnson & Johnson shot or with the other vaccines, which use a different technology?  “People should generally get the same vaccine as their initial series,” said a federal official. “But some people might not have access to their original vaccine, and some people might have had a bad reaction to the mRNA vaccines — the technology used to make the Pfizer-BioNTech and Moderna shots.”  A recent preprint study, not yet evaluated by outside experts or published in a scientific journal, provides answers. The study tested mixing booster doses from different companies The findings could be good news for the 15 million people who received the Johnson & Johnson vaccine in the United States, many of whom have felt left out because the vast majority of U.S. vaccine recipients received either the Pfizer-BioNTech or Moderna vaccine.  Data from the preprint study showed that while recipients of the Johnson & Johnson coronavirus vaccine can benefit from a second dose of the original, they may derive even greater protection if the boost comes from a different vaccine technology.  The preprint research showed that a second shot using the Moderna vaccine triggered the biggest boost of virus-neutralizing antibodies in Johnson & Johnson recipients, resulting in a 76-fold increase in antibody levels. A Pfizer booster increased antibody levels 35-fold. A matching Johnson & Johnson booster triggered only a 4-fold increase.  The take-home message from these preprint study results is that those who need to “mix and match” their booster shot and original vaccine – especially if the original was Johnson & Johnson – will still receive a protective benefit. |

**Supplemental Material 2**

***Measures***

| Variable | Question wording | Response options |
| --- | --- | --- |
| Age | *What is your age?* | 18 – 115 |
| Education | *What is the highest grade of school you have completed?* | < High school  High school/GED  Some college/associate’s degree  Four-year college degree  Advanced/professional degree |
| Preference for Information about Uncertain Science (PIUS-7)  (Ratcliff & Wicke, 2023; also see Ratcliff et al., 2023) | *Please rate how much you agree or disagree with the following statements.*   1. I like it when scientists describe the limitations of their studies, in addition to the benefits. 2. I like it when the caveats of a scientific study are fully explained. 3. I like to learn about new scientific discoveries, even if they’re too preliminary to be acted upon. 4. Science journalists should describe the uncertainties or unknowns when reporting about a scientific discovery. 5. I like to know about the limitations and caveats surrounding new research findings. 6. I like to learn about new scientific discoveries, even if they don't yet translate to solutions in the real world. 7. When learning about a new scientific discovery, I want to know how well the evidence supports a particular claim. | 1=strongly disagree  2=somewhat disagree  3=neither agree nor disagree  4=somewhat agree  5=strongly agree |
| Factual Scientific Literacy (National Science Board, 2020) | *True or False?*   1. The center of the Earth is very hot. (T) 2. The continents have been moving their location for millions of years and will continue to move. (T) 3. All radioactivity is man-made. (F) 4. Electrons are smaller than atoms. (T) 5. Lasers work by focusing sound waves. (F) 6. It is the father’s gene that decides whether the baby is a boy or a girl. (T) 7. Antibiotics kill viruses as well as bacteria. (F) | 0=inaccurate or don’t know  1=accurate  Score range: 0–7 |

**Supplemental Material 3**

***Participant characteristics by sample (with general population samples split)***

| **Variable** | **Students**  **(N=837)** | **General Population Sample 1**  **(N=433)** | **General Population Sample 2**  **(N=431)** |
| --- | --- | --- | --- |
| Age | Mean = 19.19  Range: 18–29 | Mean = 49.08  Range: 18–94 | Mean = 47.35  Range: 18–85 |
| PIUS | Mean = 4.23 (SD = .50) | Mean = 3.90 (SD = .76) | Mean = 3.82 (SD = .90) |
| Factual Scientific Literacy | Mean = 5.64 (SD = 1.14) | Mean = 5.03 (SD = 1.40) | *Not measured* |
|  | **N (%)** | **N (%)** |  |
| Education |  |  |  |
| < High school | 3 (0.4) | 31 (7.1) | 33 (7.7) |
| High school/GED | 269 (32.1) | 189 (43.5) | 180 (41.8) |
| Some college/associate’s degree | 554 (66.2) | 100 (23.0) | 113 (26.2) |
| Four-year college degree | 11 (1.3) | 70 (16.1) | 55 (12.8) |
| Advanced/professional degree | 0 | 43 (9.9) | 50 (11.6) |
| Notes. There were no statistically significant differences between General Population Samples 1 and 2 for age (*t*[862) = 1.411, *p* = .159), PIUS (*t*[862] = 1.316, *p* = .189), or education (*t*[862) = –.108, *p* = .914). Factual Scientific Literacy was not measured in Sample 2. | | | |

**Supplemental Material 4**

***Codebook and Sample Responses for Deductive Content Analysis of Open-ended Data***

| **Code** | | **Definition** | **Examples** |
| --- | --- | --- | --- |
| A | Not peer reviewed | Refers to study not being reviewed by peers, experts, scientists, a scientific journal, editor, etc. Can refer to it as being “unreviewed,” “not peer reviewed,” “unverified by peers/experts”* or a synonymous term.  **Must state ‘unverified by peer review’ or synonymous; ‘unverified by subsequent research/testing’ is counted as D.*  “Fact checking” (or editing or proofreading) not counted here unless they specified as “by scientists/researchers” to distinguish from journalistic fact-checking/editing/proofreading. | “it hasn’t been fully vetted by experts.” (1-314)  “It’s an article shared before peer review” (2-261)  “I think that preprint means that it has not undergone scrutiny and cannot fully be trusted yet. Preprint has not been scientifically reviewed and cannot be inferred to be 100% accurate.” (3-421) |
| B | Released publicly | Refers to study/paper being posted (or “released,” “shared,” “published,” “uploaded,” etc.) publicly or on a preprint server. Response must specifically reference being shared with the public or on a preprint server.  *Note: Our original category definition specified that the research is posted by the scientists/authors; however, there were no instances of this in our prior coding, so we broadened the definition.* | “draft shared publicly before shared with peers.” (1-312)  “something is being printed to the public before the study has been officially posted.” (3-592) |
| C | Not published in a scientific journal | Refers to study not being published in a scientific journal (or official outlet, academic journal, scientific publication, etc.). Must specify something like “outlet” or “journal” … if they just say “not officially published” count it as Other. | “It has not been published in a journal but is being released to show what is being done.” (1-242)  “that it hasn’t been published in a medical journal yet.” (2-407) |
| D | Preliminary or not final results (i.e., uncertain evidence) | Refers to the research (findings/evidence/results) as being unverified, unvalidated, unproven, inconclusive, unconfirmed, unauthenticated, not established, not known for sure, etc.; says “conclusions not final”, “more research needed”, not all the facts are in, not proven conclusively, “preliminary evidence,” or “inconclusive research.” If not in clear reference to the research results (e.g., references to “information,” “sources,” “article,” or “it”), code as Other.  If they say the study (or line of studies/research) has not been completed, code here. However, if they say the study hasn’t been started (e.g., “article came before the study”), code as Other.  Count reference to the state of the evidence for any topic participants were exposed to a message about in the study (e.g., Mardi Gras super spreader event, booster vaccines, etc) | “A paper that hasn’t been thoroughly peer reviewed and subject to errors.” (1-276)  “It’s being talked about in print before the study is complete.” (2-169)  “The article was printed before any of the data has been confirmed by science.” (3-175) |
| E | Other* | Relevant responses that don’t fit into the other categories.  Includes simply saying “not published (yet),” “not printed (yet),” “already printed,” “not available (yet); Also includes descriptions of the article/writing as “not final” (preliminary draft, early draft, not final version or draft, “rough draft,” “working” draft/article, unfinished, etc.), not [“officially” or “formally”] published/printed [yet].^[[1]](#footnote-1)^  *Treated as a mutually exclusive category. Only code as other if no part of a response fits into another category. | “Easier for the reader to understand.” (1-35)  “It is preliminary but generally agreed upon so almost ready to go into full printing” (2-369)  “That is not yet ready for public for fact checking reasons.” (3-40) |
| F | Doesn’t know | Says they’re “unsure,” “don’t know,” “IDK,” “not familiar with the term,” or something similar. | “I don’t know what preprint refers to.” (2-179)  “Nothing I have no idea what it is for.” (3-43) |
| G | Blank/ irrelevant response | Participant gave no response or an irrelevant response (e.g., descriptions that have nothing to do with research, publishing, peer review, etc.).  Include non-English responses and gibberish responses here. Also include -99 and ambiguous responses here. | “na.” (2-371)  “nothing.” (1-334) |

***Codebook and Sample Responses for Inductive Content Analysis of Open-ended Data***

| **Theme** | **Definition** | **Examples** |
| --- | --- | --- |
| Printing and publication | Defines *preprint* in relation to the printing and publication process, without referencing academic journal publishing. Includes definitions that conceptualize preprints as:   - Previews or introductions to content that will be published in the future; - Copies, reprints, or rewritten versions of content that had been printed or published previously; - Unpublished or partially published content. | “Before it has been published or will be published” (1-194)  “...a precursor to a larger, more expansive article regarding the same topic” (3-224)  “It was already written and someone copied it” (1-55)  “Not a fully published article” (1-92) |
| Uncertain and incomplete information | Defines *preprint* as information that is unfinished, unofficial, unverified, initial, or otherwise incomplete, without specifying that the information is research. Includes definitions that conceptualize this uncertainty as suspicious or problematic as well as those that are unphased by the uncertain state of the information. | “To be honest it can make me uncertain about how effective a preprint will be because it seems less official” (3-555)  “the article is not yet ready to be published but it is on the right track to being 100% correct and ready” (3-357) |
| (Scientific) news stories | Defines *preprint* as a news story, typically one focused on science. Definitions may describe the story as not fully proofread, edited, fact checked, written, “peer reviewed,” or approved for print, or as being unofficial in some other way. | “It is an article that is composed with basic facts that can be changed before it is made for print in national or local newspapers” (1-394)  “It was not printed yet onto news articles for the general public” (3-307) |
| Complete and credible information | Defines *preprint* as a confirmed and credible source of information. Includes definitions that demonstrate a misunderstanding of preprints (e.g., describing them as peer reviewed or verified studies) as well as claims that the print or publication status of the information does not impact its accuracy or trustworthiness. | “...after the scientific peer evaluation but it still needs to be published” (3-342)  “A reliable article that has not been published yet” (2-283) |

**Supplemental Material 5**

***Preprint Definitions by Experimental Message Version***

**5a. General Population Sample 1 (N = 433)**

**Results by condition, with brief preprint disclosure and expanded preprint disclosure conditions separated.**

*Crosstabs Output*

| **Participant defined preprint as A, B and/or C** | | | | | |
| --- | --- | --- | --- | --- | --- |
| Count | | | | | |
|  | | Condition | | | Total |
|  |  | Control | Preprint Brief Disclosure | Preprint Expanded Disclosure |  |
| Response coded as A, B and/or C | No (0) | 135_a_ | 131_a_ | 117_a_ | 383 |
|  | Yes (1) | 12_a_ | 17_a_ | 21_a_ | 50 |
| Total | | 147 | 148 | 138 | 433 |
| Note. Each subscript letter denotes a subset of Condition categories whose column proportions do not differ significantly from each other at the .05 level. | | | | | |

| **Chi-Square Tests** | | | |
| --- | --- | --- | --- |
|  | Value | df | Asymptotic Significance (2-sided) |
| Pearson Chi-Square | 3.469^a^ | 2 | .177 |
| Likelihood Ratio | 3.489 | 2 | .175 |
| Linear-by-Linear Association | 3.457 | 1 | .063 |
| N of Valid Cases | 433 |  |  |
| a. 0 cells (.0%) have expected count less than 5. The minimum expected count is 15.94. | | | |

| **Participant defined preprint as D** | | | | | | | | | |
| --- | --- | --- | --- | --- | --- | --- | --- | --- | --- |
| Count | | | | | | | | | |
|  | | Condition | | | | | | Total | |
|  |  | Control (No Preprint Disclosure) | | Preprint Brief Disclosure | | Preprint Expanded Disclosure | |  |  |
| Response coded as D | No (0) | 124_a_ | | 116_a_ | | 105_a_ | | 345 | |
|  | Yes (1) | 23_a_ | | 32_a_ | | 33_a_ | | 88 | |
| Total | | 147 | | 148 | | 138 | | 433 | |
| Each subscript letter denotes a subset of Condition categories whose column proportions do not differ significantly from each other at the .05 level. | | | | | | | | | |
| **Chi-Square Tests** | | | | | | | | |  |
|  | | | Value | | df | | Asymptotic Significance (2-sided) | |  |
| Pearson Chi-Square | | | 3.238^a^ | | 2 | | .198 | |  |
| Likelihood Ratio | | | 3.322 | | 2 | | .190 | |  |
| Linear-by-Linear Association | | | 3.027 | | 1 | | .082 | |  |
| N of Valid Cases | | | 433 | |  | |  | |  |
| a. 0 cells (.0%) have expected count less than 5. The minimum expected count is 28.05. | | | | | | | | |  |

------------------------------------------------------------------------------------

**Results by condition, with brief and expanded preprint disclosure conditions combined.**

| **Participant defined preprint as A, B and/or C** | | | | |
| --- | --- | --- | --- | --- |
| Count | | | | |
|  | | Condition | | Total |
|  |  | No disclosure | Preprint disclosure |  |
| Response coded as A, B and/or C | No (0) | 133_a_ | 255_a_ | 388 |
|  | Yes (1) | 14_a_ | 31_a_ | 45 |
| Total | | 147 | 286 | 433 |
| Each subscript letter denotes a subset of No disclosure vs Either disclosure categories whose column proportions do not differ significantly from each other at the .05 level. | | | | |

| **Chi-Square Tests** | | | | | |
| --- | --- | --- | --- | --- | --- |
|  | Value | df | Asymptotic Significance (2-sided) | Exact Sig. (2-sided) | Exact Sig. (1-sided) |
| Pearson Chi-Square | .180^a^ | 1 | .671 |  |  |
| Continuity Correction^b^ | .067 | 1 | .796 |  |  |
| Likelihood Ratio | .183 | 1 | .669 |  |  |
| Fisher's Exact Test |  |  |  | .741 | .403 |
| Linear-by-Linear Association | .180 | 1 | .671 |  |  |
| N of Valid Cases | 433 |  |  |  |  |
| a. 0 cells (0.0%) have expected count less than 5. The minimum expected count is 15.28. | | | | | |
| b. Computed only for a 2x2 table | | | | | |

| **Participant defined preprint as D** | | | | |
| --- | --- | --- | --- | --- |
| Count | | | | |
|  | | Condition | | Total |
|  |  | No disclosure | Preprint disclosure |  |
| Response coded as D | No (0) | 140_a_ | 264_a_ | 404 |
|  | Yes (1) | 7_a_ | 22_a_ | 29 |
| Total | | 147 | 286 | 433 |
| Each subscript letter denotes a subset of No disclosure vs Either disclosure categories whose column proportions do not differ significantly from each other at the .05 level. | | | | |

| **Chi-Square Tests** | | | | | |
| --- | --- | --- | --- | --- | --- |
|  | Value | df | Asymptotic Significance (2-sided) | Exact Sig. (2-sided) | Exact Sig. (1-sided) |
| Pearson Chi-Square | 1.334^a^ | 1 | .248 |  |  |
| Continuity Correction^b^ | .907 | 1 | .341 |  |  |
| Likelihood Ratio | 1.408 | 1 | .235 |  |  |
| Fisher's Exact Test |  |  |  | .312 | .171 |
| Linear-by-Linear Association | 1.331 | 1 | .249 |  |  |
| N of Valid Cases | 433 |  |  |  |  |
| a. 0 cells (0.0%) have expected count less than 5. The minimum expected count is 9.85. | | | | | |
| b. Computed only for a 2x2 table | | | | | |

**5b. General Population Sample 2 (N = 433)**

| **Participant defined preprint as A, B and/or C** | | | | |
| --- | --- | --- | --- | --- |
| Count | | | | |
|  | | Condition | | Total |
|  |  | No disclosure | Preprint disclosure |  |
| Response coded as A, B and/or C | No (0) | 171_a_ | 214_a_ | 385 |
|  | Yes (1) | 20_a_ | 26_a_ | 46 |
| Total | | 191 | 240 | 431 |
| Each subscript letter denotes a subset of Preprint understanding coded as A-C (1 = yes) categories whose column proportions do not differ significantly from each other at the .05 level. | | | | |

| **Chi-Square Tests** | | | | | |
| --- | --- | --- | --- | --- | --- |
|  | Value | df | Asymptotic Significance (2-sided) | Exact Sig. (2-sided) | Exact Sig. (1-sided) |
| Pearson Chi-Square | .015^a^ | 1 | .904 |  |  |
| Continuity Correction^b^ | .000 | 1 | 1.000 |  |  |
| Likelihood Ratio | .015 | 1 | .904 |  |  |
| Fisher's Exact Test |  |  |  | 1.000 | .516 |
| Linear-by-Linear Association | .015 | 1 | .904 |  |  |
| N of Valid Cases | 431 |  |  |  |  |
| a. 0 cells (0.0%) have expected count less than 5. The minimum expected count is 20.39. | | | | | |
| b. Computed only for a 2x2 table | | | | | |

| **Participant defined preprint as D** | | | | |
| --- | --- | --- | --- | --- |
| Count | | | | |
|  | | Condition | | Total |
|  |  | No disclosure | Preprint disclosure |  |
| Response coded as D | No (0) | 174_a_ | 226_a_ | 400 |
|  | Yes (1) | 17_a_ | 14_a_ | 31 |
| Total | | 191 | 240 | 431 |
| Each subscript letter denotes a subset of D categories whose column proportions do not differ significantly from each other at the .05 level. | | | | |

| **Chi-Square Tests** | | | | | |
| --- | --- | --- | --- | --- | --- |
|  | Value | df | Asymptotic Significance (2-sided) | Exact Sig. (2-sided) | Exact Sig. (1-sided) |
| Pearson Chi-Square | 1.499^a^ | 1 | .221 |  |  |
| Continuity Correction^b^ | 1.075 | 1 | .300 |  |  |
| Likelihood Ratio | 1.487 | 1 | .223 |  |  |
| Fisher's Exact Test |  |  |  | .261 | .150 |
| Linear-by-Linear Association | 1.495 | 1 | .221 |  |  |
| N of Valid Cases | 431 |  |  |  |  |
| a. 0 cells (0.0%) have expected count less than 5. The minimum expected count is 13.74. | | | | | |
| b. Computed only for a 2x2 table | | | | | |

**5c. Combined General Population Samples (Samples 1 and 2; N = 864)**

Note: There were no significant differences between the two general population samples with respect to the accuracy of their preprint definitions (see table below). As such, for simplicity, we combined them into a single general population sample (*n*=864).

***Differences in preprint understanding between General Population Samples 1 and 2***

| **Variable** | **General Population Sample 1**  **(N=433)** | **General Population Sample 2**  **(N=431)** |
| --- | --- | --- |
| Participant defined preprint as A, B and/or C | n=45 | n=46 |
|  | Difference: *t*(862) = –.134, *p* = .893 | |
| Participant defined preprint as D | n=29 | n=31 |
|  | Difference: *t*(862) = –.286, *p* = .775 | |

Note: Sample sizes are unequal between the two comparison groups (i.e., N=338 vs N=526) because the two preprint disclosure conditions in Sample 1 were merged. Therefore, it is important to compare the bolded percentages rather than the counts per group.

| **Participant defined preprint as A, B, and/or C** | | | | | |
| --- | --- | --- | --- | --- | --- |
|  | | | Condition | | Total |
|  |  |  | No preprint disclosure | Preprint disclosure |  |
| Response coded as A, B, and/or C | No (0) | Count | 304_a_ | 469_a_ | 773 |
|  |  | % within Preprint understanding coded as A-C (1 = yes) | 39.3% | 60.7% | 100.0% |
|  |  | % within Preprint disclosure condition | **89.9%** | **89.2%** | 89.5% |
|  |  | % of Total | 35.2% | 54.3% | 89.5% |
|  | Yes (1) | Count | 34_a_ | 57_a_ | 91 |
|  |  | % within Preprint understanding coded as A-C (1 = yes) | 37.4% | 62.6% | 100.0% |
|  |  | % within Preprint disclosure condition | **10.1%** | **10.8%** | 10.5% |
|  |  | % of Total | 3.9% | 6.6% | 10.5% |
| Total | | Count | **338** | **526** | **864** |
|  |  | % within Preprint understanding coded as A-C (1 = yes) | 39.1% | 60.9% | 100.0% |
|  |  | % within Preprint disclosure condition | 100.0% | 100.0% | 100.0% |
|  |  | % of Total | 39.1% | 60.9% | 100.0% |
| Each subscript letter denotes a subset of Preprint 1 and 3 combined (0=no preprint, 1=preprint) categories whose column proportions do not differ significantly from each other at the .05 level. | | | | | |

| **Chi-Square Tests** | | | | | |
| --- | --- | --- | --- | --- | --- |
|  | Value | df | Asymptotic Significance (2-sided) | Exact Sig. (2-sided) | Exact Sig. (1-sided) |
| Pearson Chi-Square | .132^a^ | 1 | .716 |  |  |
| Continuity Correction^b^ | .062 | 1 | .803 |  |  |
| Likelihood Ratio | .133 | 1 | .716 |  |  |
| Fisher's Exact Test |  |  |  | .735 | .404 |
| Linear-by-Linear Association | .132 | 1 | .717 |  |  |
| N of Valid Cases | 864 |  |  |  |  |
| a. 0 cells (.0%) have expected count less than 5. The minimum expected count is 35.60. | | | | | |
| b. Computed only for a 2x2 table | | | | | |

| **Participant defined preprint as D** | | | | | |
| --- | --- | --- | --- | --- | --- |
|  | | | Condition | | Total |
|  |  |  | No preprint disclosure | Preprint disclosure |  |
| Response coded as D | No (0) | Count | 314_a_ | 490_a_ | 804 |
|  |  | % within D | 39.1% | 60.9% | 100.0% |
|  |  | % within Preprint disclosure condition | 92.9% | 93.2% | 93.1% |
|  |  | % of Total | 36.3% | 56.7% | 93.1% |
|  | Yes (1) | Count | 24_a_ | 36_a_ | 60 |
|  |  | % within D | 40.0% | 60.0% | 100.0% |
|  |  | % within Preprint disclosure condition | 7.1% | 6.8% | 6.9% |
|  |  | % of Total | 2.8% | 4.2% | 6.9% |
| Total | | Count | 338 | 526 | 864 |
|  |  | % within D | 39.1% | 60.9% | 100.0% |
|  |  | % within Preprint disclosure condition | 100.0% | 100.0% | 100.0% |
|  |  | % of Total | 39.1% | 60.9% | 100.0% |
| Each subscript letter denotes a subset of Preprint 1 and 3 combined (0=no preprint, 1=preprint) categories whose column proportions do not differ significantly from each other at the .05 level. | | | | | |

| **Chi-Square Tests** | | | | | |
| --- | --- | --- | --- | --- | --- |
|  | Value | df | Asymptotic Significance (2-sided) | Exact Sig. (2-sided) | Exact Sig. (1-sided) |
| Pearson Chi-Square | .021^a^ | 1 | .885 |  |  |
| Continuity Correction^b^ | .000 | 1 | .994 |  |  |
| Likelihood Ratio | .021 | 1 | .885 |  |  |
| Fisher's Exact Test |  |  |  | .892 | .494 |
| Linear-by-Linear Association | .021 | 1 | .885 |  |  |
| N of Valid Cases | 864 |  |  |  |  |
| a. 0 cells (.0%) have expected count less than 5. The minimum expected count is 23.47. | | | | | |
| b. Computed only for a 2x2 table | | | | | |

**5d. Student Sample (N=837)**

| **Participant defined preprint as A, B and/or C** | | | | |
| --- | --- | --- | --- | --- |
| Count | | | | |
|  | | Condition | | Total |
|  |  | No disclosure | Preprint disclosure |  |
| Response coded as A, B and/or C | No (0) | 323_a_ | 279_a_ | 602 |
|  | Yes (1) | 99_b_ | 136_b_ | 235 |
| Total | | 422 | 415 | 837 |
| Each subscript letter denotes a subset of Preprint understanding coded as A-C (1 = yes) categories whose column proportions do not differ significantly from each other at the .05 level. | | | | |

| **Chi-Square Tests** | | | | | |
| --- | --- | --- | --- | --- | --- |
|  | Value | df | Asymptotic Significance (2-sided) | Exact Sig. (2-sided) | Exact Sig. (1-sided) |
| Pearson Chi-Square | 8.984^a^ | 1 | .003 |  |  |
| Continuity Correction^b^ | 8.528 | 1 | .003 |  |  |
| Likelihood Ratio | 9.010 | 1 | .003 |  |  |
| Fisher's Exact Test |  |  |  | .003 | .002 |
| Linear-by-Linear Association | 8.973 | 1 | .003 |  |  |
| N of Valid Cases | 837 |  |  |  |  |
| a. 0 cells (0.0%) have expected count less than 5. The minimum expected count is 116.52. | | | | | |
| b. Computed only for a 2x2 table | | | | | |

| **Participant defined preprint as D** | | | | |
| --- | --- | --- | --- | --- |
| Count | | | | |
|  | | Condition | | Total |
|  |  | No disclosure | Preprint disclosure |  |
| Response coded as D | No (0) | 360_a_ | 350_a_ | 710 |
|  | Yes (1) | 62_a_ | 65_a_ | 127 |
| Total | | 422 | 415 | 837 |
| Each subscript letter denotes a subset of Preprint understanding coded as D (1 = yes) categories whose column proportions do not differ significantly from each other at the .05 level. | | | | |

| **Chi-Square Tests** | | | | | |
| --- | --- | --- | --- | --- | --- |
|  | Value | df | Asymptotic Significance (2-sided) | Exact Sig. (2-sided) | Exact Sig. (1-sided) |
| Pearson Chi-Square | .153^a^ | 1 | .696 |  |  |
| Continuity Correction^b^ | .087 | 1 | .768 |  |  |
| Likelihood Ratio | .153 | 1 | .696 |  |  |
| Fisher's Exact Test |  |  |  | .701 | .384 |
| Linear-by-Linear Association | .153 | 1 | .696 |  |  |
| N of Valid Cases | 837 |  |  |  |  |
| a. 0 cells (0.0%) have expected count less than 5. The minimum expected count is 62.97. | | | | | |
| b. Computed only for a 2x2 table | | | | | |

**Supplemental Material 6**

***Differences in Preprint Understanding and Individual Characteristics Between Student and General Population Samples***

| **Variable** | **Students**  **(*n*=837)** | **General Population**  **(*n*=864)** | **Difference** |
| --- | --- | --- | --- |
| Age | Mean = 19.19 (SD = 1.26) | Mean = 48.22 (SD = 18.03) | *t*(1699) = 46.457, *p* < .001 |
| PIUS | Mean = 4.23  (SD = .50) | Mean = 3.86  (SD = .83) | *t*(1699) = –11.185, *p* < .001 |
| Factual Scientific Literacy | Mean = 5.64  (SD = 1.14) | Mean = 5.03 (SD = 1.40) | *t*(1265) = –8.471, *p* < .001 |
| Education (Highest grade completed) | Mean = 14.73 (SD = .59) | Mean = 15.01 (SD = 1.90) | *t*(1699) = 4.154, *p* < .001 |
| Participant defined preprint as A, B and/or C | Count: 235 (28%)  Mean = .28  (SD = .45) | Count: 91 (11%)  Mean = .11 (SD = .31) | *t*(1699) = –9.422, *p* < .001 |
| Participant defined preprint as D | Count: 127 (15%)  Mean = .15 (SD = .36) | Count: 60 (7%)  Mean = .07 (SD = .25) | *t*(1699) = –5.468, *p* < .001 |

**Supplemental Material 7**

***Individual Difference Correlates of Defining Preprint as Preliminary***

|  | Students | General Population |
| --- | --- | --- |
| Age | -.04 | .00 |
| Education |  | .01 |
| PIUS | -.01 | .09** |
| Factual Scientific Literacy | -.04 | .04^✝^ |
| *Notes*. * *p* <.05, ** *p* <.01, *** *p* <.001. Defining preprint as preliminary corresponds to code D. ^✝^Correlation for Factual Scientific Literacy based only on data from the Student Sample and General Population Sample in Study 1, as it was not measured for the General Population Sample in Study 2. No correlation was reported for students’ education because all students had the same numeric value for education as measured. | | |

1. We treated such responses as ‘Other’ because they were too ambiguous to code as one of our predefined categories; e.g., they could be referring to publication of the news article rather than publication in a scientific journal. [↑](#footnote-ref-1)
